# Supplementary material for: HIVprotI: an integrated web based platform for prediction and design of HIV proteins inhibitors
Source: J Cheminform. 2018 Mar 9;10:12. doi: 10.1186/s13321-018-0266-y (PMC5845081; doi:10.1186/s13321-018-0266-y)
Supplement: Supplementary file 2 — Additional file 2. Source code of HIVProtI web server. [file 13321_2018_266_MOESM2_ESM.zip › HIVprotI_Source-code/hivprotI/weka-3-6-6/doc/weka/core/converters/XRFFLoader.html]

XRFFLoader


---


|  |  |  |
| --- | --- | --- |
| **Overview** \| **Package** \| **Class** \| **Tree** \| **Deprecated** \| **Index** \| **Help** \| **Weka's home** | |  |
| **PREV CLASS**   **NEXT CLASS** | **FRAMES**    **NO FRAMES**     **All Classes** |  |
| SUMMARY: NESTED \| FIELD \| CONSTR \| METHOD | DETAIL: FIELD \| CONSTR \| METHOD |  |


---


## weka.core.converters Class XRFFLoader

```
java.lang.Object
  weka.core.converters.AbstractLoader
      weka.core.converters.AbstractFileLoader
          weka.core.converters.XRFFLoader
```

**All Implemented Interfaces:**: java.io.Serializable, BatchConverter, FileSourcedConverter, Loader, URLSourcedLoader, EnvironmentHandler, RevisionHandler

---

``` public class XRFFLoader extends AbstractFileLoader implements BatchConverter, URLSourcedLoader ```

Reads a source that is in the XML version of the ARFF format. It automatically decompresses the data if the extension is '.xrff.gz'.

**Version:**
:   $Revision: 4985 $

**Author:**
:   FracPete (fracpete at waikato dot ac dot nz)

**See Also:**: `Loader`, Serialized Form

---

| **Field Summary** | |
| --- | --- |
| `static java.lang.String` | `FILE_EXTENSION`             the file extension |
| `static java.lang.String` | `FILE_EXTENSION_COMPRESSED`             the extension for compressed files |

**Fields inherited from interface weka.core.converters.Loader**

`BATCH, INCREMENTAL, NONE`


**Constructor Summary**

`XRFFLoader()`


| **Method Summary** | |
| --- | --- |
| `Instances` | `getDataSet()`             Return the full data set. |
| `java.lang.String` | `getFileDescription()`             Returns a description of the file type. |
| `java.lang.String` | `getFileExtension()`             Get the file extension used for libsvm files |
| `java.lang.String[]` | `getFileExtensions()`             Gets all the file extensions used for this type of file |
| `Instance` | `getNextInstance(Instances structure)`             XRFFLoader is unable to process a data set incrementally. |
| `java.lang.String` | `getRevision()`             Returns the revision string. |
| `Instances` | `getStructure()`             Determines and returns (if possible) the structure (internally the header) of the data set as an empty set of instances. |
| `java.lang.String` | `globalInfo()`             Returns a string describing this Loader |
| `static void` | `main(java.lang.String[] args)`             Main method. |
| `void` | `reset()`             Resets the Loader ready to read a new data set |
| `java.lang.String` | `retrieveURL()`             Return the current url |
| `void` | `setSource(java.io.File file)`             Resets the Loader object and sets the source of the data set to be the supplied File object. |
| `void` | `setSource(java.io.InputStream in)`             Resets the Loader object and sets the source of the data set to be the supplied InputStream. |
| `void` | `setSource(java.net.URL url)`             Resets the Loader object and sets the source of the data set to be the supplied url. |
| `void` | `setURL(java.lang.String url)`             Set the url to load from |

**Methods inherited from class weka.core.converters.AbstractFileLoader**

`getUseRelativePath, retrieveFile, runFileLoader, setEnvironment, setFile, setUseRelativePath, useRelativePathTipText`

**Methods inherited from class weka.core.converters.AbstractLoader**

`setRetrieval`

**Methods inherited from class java.lang.Object**

`equals, getClass, hashCode, notify, notifyAll, toString, wait, wait, wait`

**Field Detail**

### FILE\_EXTENSION

```
public static java.lang.String FILE_EXTENSION
```

:   the file extension

---


### FILE\_EXTENSION\_COMPRESSED

```
public static java.lang.String FILE_EXTENSION_COMPRESSED
```

:   the extension for compressed files


**Constructor Detail**

### XRFFLoader

```
public XRFFLoader()
```


**Method Detail**

### globalInfo

```
public java.lang.String globalInfo()
```

:   Returns a string describing this Loader

    :   **Returns:**: a description of the Loader suitable for displaying in the explorer/experimenter gui

---


### getFileExtension

```
public java.lang.String getFileExtension()
```

:   Get the file extension used for libsvm files

    :   **Specified by:**: `getFileExtension` in interface `FileSourcedConverter`
    :   **Returns:**: the file extension

---


### getFileExtensions

```
public java.lang.String[] getFileExtensions()
```

:   Gets all the file extensions used for this type of file

    :   **Specified by:**: `getFileExtensions` in interface `FileSourcedConverter`
    :   **Returns:**: the file extensions

---


### getFileDescription

```
public java.lang.String getFileDescription()
```

:   Returns a description of the file type.

    :   **Specified by:**: `getFileDescription` in interface `FileSourcedConverter`
    :   **Returns:**: a short file description

---


### reset

```
public void reset()
           throws java.io.IOException
```

:   Resets the Loader ready to read a new data set

    :   **Specified by:**: `reset` in interface `Loader` **Overrides:**: `reset` in class `AbstractFileLoader`
    :   **Throws:**: `java.io.IOException` - if something goes wrong

---


### setSource

```
public void setSource(java.io.File file)
               throws java.io.IOException
```

:   Resets the Loader object and sets the source of the data set to be
    the supplied File object.

    :   **Specified by:**: `setSource` in interface `Loader` **Overrides:**: `setSource` in class `AbstractFileLoader`
    :   **Parameters:**: `file` - the source file. **Throws:**: `java.io.IOException` - if an error occurs

---


### setSource

```
public void setSource(java.net.URL url)
               throws java.io.IOException
```

:   Resets the Loader object and sets the source of the data set to be
    the supplied url.

    :   **Parameters:**: `url` - the source url. **Throws:**: `java.io.IOException` - if an error occurs

---


### setURL

```
public void setURL(java.lang.String url)
            throws java.io.IOException
```

:   Set the url to load from

    :   **Specified by:**: `setURL` in interface `URLSourcedLoader`
    :   **Parameters:**: `url` - the url to load from **Throws:**: `java.io.IOException` - if the url can't be set.

---


### retrieveURL

```
public java.lang.String retrieveURL()
```

:   Return the current url

    :   **Specified by:**: `retrieveURL` in interface `URLSourcedLoader`
    :   **Returns:**: the current url

---


### setSource

```
public void setSource(java.io.InputStream in)
               throws java.io.IOException
```

:   Resets the Loader object and sets the source of the data set to be
    the supplied InputStream.

    :   **Specified by:**: `setSource` in interface `Loader` **Overrides:**: `setSource` in class `AbstractLoader`
    :   **Parameters:**: `in` - the source InputStream. **Throws:**: `java.io.IOException` - if initialization of reader fails.

---


### getStructure

```
public Instances getStructure()
                       throws java.io.IOException
```

:   Determines and returns (if possible) the structure (internally the
    header) of the data set as an empty set of instances.

    :   **Specified by:**: `getStructure` in interface `Loader` **Specified by:**: `getStructure` in class `AbstractLoader`
    :   **Returns:**: the structure of the data set as an empty set of Instances **Throws:**: `java.io.IOException` - if an error occurs

---


### getDataSet

```
public Instances getDataSet()
                     throws java.io.IOException
```

:   Return the full data set. If the structure hasn't yet been determined
    by a call to getStructure then method should do so before processing
    the rest of the data set.

    :   **Specified by:**: `getDataSet` in interface `Loader` **Specified by:**: `getDataSet` in class `AbstractLoader`
    :   **Returns:**: the structure of the data set as an empty set of Instances **Throws:**: `java.io.IOException` - if there is no source or parsing fails

---


### getNextInstance

```
public Instance getNextInstance(Instances structure)
                         throws java.io.IOException
```

:   XRFFLoader is unable to process a data set incrementally.

    :   **Specified by:**: `getNextInstance` in interface `Loader` **Specified by:**: `getNextInstance` in class `AbstractLoader`
    :   **Parameters:**: `structure` - ignored **Returns:**: never returns without throwing an exception **Throws:**: `java.io.IOException` - always. XRFFLoader is unable to process a data set incrementally.

---


### getRevision

```
public java.lang.String getRevision()
```

:   Returns the revision string.

    :   **Specified by:**: `getRevision` in interface `RevisionHandler`
    :   **Returns:**: the revision

---


### main

```
public static void main(java.lang.String[] args)
```

:   Main method.

    :   **Parameters:**: `args` - should contain the name of an input file.


---


|  |  |  |
| --- | --- | --- |
| **Overview** \| **Package** \| **Class** \| **Tree** \| **Deprecated** \| **Index** \| **Help** \| **Weka's home** | |  |
| **PREV CLASS**   **NEXT CLASS** | **FRAMES**    **NO FRAMES**     **All Classes** |  |
| SUMMARY: NESTED \| FIELD \| CONSTR \| METHOD | DETAIL: FIELD \| CONSTR \| METHOD |  |


---
